# Antinociceptive and cytotoxic activities of an epiphytic medicinal orchid: *Vanda tessellata* Roxb.

**DOI:** 10.1186/1472-6882-14-464

**Published:** 2014-12-03

**Authors:** M Anisuzzaman Chowdhury, M Masudur Rahman, Mohammed Riaz Hasan Chowdhury, M Josim Uddin, Mohammed Abu Sayeed, M Aslam Hossain

**Affiliations:** Department of Pharmacy, Faculty of Science and Engineering, International Islamic University Chittagong, 154/A, College Road, Chittagong, 4203 Bangladesh; Department of Pharmaceutical Chemistry, Faculty of Pharmacy, University of Dhaka, Dhaka, 1000 Bangladesh

**Keywords:** Antinociceptive, Analgesic, Tail immersion, hot plate, Cytotoxicity, *Vanda tessellata*

## Abstract

**Background:**

*Vanda tessellata* (Orchidaceae) has been used in different sorts of ailments such as inflammations, rheumatism, dysentery, bronchitis, dyspepsia and fever in folk medicine. In this study we evaluated the antinociceptive and cytotoxic effect of methanol and aqueous extracts of *V. tessellata* leaf.

**Methods:**

Oral administration of *V. tessellata* aqueous (VTA) and methanol (VTM) leaf extracts at 200 and 400 mg/kg body weight (bw) doses were assessed for antinociceptive activity in acetic acid-induced writhing test, hot plate test, and tail immersion test in mice. In this study we also screened for cytotoxicity of the extracts by the brine shrimp (*Artemia salina*) lethality assay.

**Results:**

The extracts, at both doses, exhibited a significant (p < 0.05 - < 0.01) dose-dependent antinociceptive activity in hot plate and tail immersion test. The reaction time was increased to the thermal stimuli at 200 and 400 mg/kg doses up to 90 min. In acetic acid-induced writhing test, oral administration of VTA and VTM (200 and 400 mg/kg) also decreased the writhing significantly while compared to control. The dose 400 mg/kg showed maximum percentage of pain inhibition 42.37% and 45.08% for VTA and VTM respectively. Diclofenac sodium (10 mg/kg) and nalbuphine (10 mg/kg) were used as reference antinociceptive drugs. Very low cytotoxicity was observed on brine shrimps lethality assay.

**Conclusions:**

The leaf extract has potential antinociceptive activity with minimum cytotoxicity. The present study supports the use of *V. tessellata* in different inflammatory disorders.

## Background

Most analgesic drugs such as NSAIDs, COX-2 inhibitors and opioids exhibit an extensive range of adverse effects including gastrointestinal disorders, kidney problems and other unwanted effects. Drug regulatory authorities have been imposed a boxed warning on the label of some COX-2 selective inhibitor for cardiovascular and gastrointestinal risks [1]. Similarly, addiction and misuse of opioids is a growing problem [2]. Analgesic choice is also determined by the type of pain, for example, traditional analgesics are less effective in neuropathic pain [3]. Therefore, advent for safe and effective analgesic drugs is still challenging for the researchers.

Nowadays, natural products as an alternative and complementary medicine to many ailments have been a major interest among researchers. In addition to documenting the traditional knowledge related to medicinal plants, scientific authentication of these medicinal plants has been an important path of recent research [4]. *Vanda tessellata* Roxb. (Orchidaceae), locally known as Rasna, has been used in folk medicine for its multifarious medicinal properties [5]. It is an epiphytic perennial herb, scandent by the stout and having simple or branching aerial roots distributed throughout Bangladesh, Indian subcontinent and Indochina. Leaves are succulent, 15–20 cm long, linear, recurved and complicate. Roots are alexiteric and antipyretic and useful in dyspepsia, bronchitis, diseases of the abdomen, bronchitis, piles, hiccough and tremors [6]. Externally the root is used in rheumatism and allied disorders and diseases of the nervous system. It is a great panacea for oligomenorrhea, amenorrhea and dysmenorrheal, as it augments uterine contractions. It is also remedy for secondary syphilis and scorpion sting. In addition, juice of the leaves is given in otitis and the paste as febrifuge. Unani practitioners hold it to be laxative and tonic to the liver. It is also used to treat boils on the scalp [7, 8]. *V. tessellata* leaves are used in folk medicine for the treatment of cough, edema, respiratory difficulties, poisoning of blood due to rheumatism, fever and paralysis in Bangladesh. Leaves of *V. tessellata* are macerated with ginger slices (rhizomes of *Zingiber officinale* Roscoe) and applied to affected areas and that application for a long time gives good results in paralysis and is satisfactory for rheumatic pain [9–11].

Experimentally, various pharmacological properties of *V. tessellata* have been reported, in particular anti-inflammatory [6, 12], antimicrobial [13], anticonvulsant [14, 15], wound healing [16], antioxidant [17], hepatoprotective [18], antidiarrheal [19], mast cell stabilization [20], cerebral anti-ischemic [21] and aphrodisiac [22, 23] properties. Phytochemical screening of the plant has revealed an alkaloid, a glucoside, tannins, β-sitosterol, γ-sitosterol and a long chain aliphatic compound, fatty oils, resins and colouring matters. Roots contain tetracosyl ferrulate and β-sitosterol-D-glucoside [7, 24]. Some bioactive compounds have been isolated from *V. tessellata* are 2,7,7-tri methyl bicyclo [2.2.1] heptanes [23], 17-β-hydroxy-14,20-epoxy-1-oxo-[22R]-3β-[O-β-d-glucopyranosyl]-5,24-withadienolide [25], and melianin [13].

The most frequent approach to species selection for phytochemical and/or pharmacological analysis is by reviewing the ethnobotanical literature [26]. In view of this evidence from the existing literatures showing that *V. tessellata* has variegated traditional uses against different sorts of diseases. Thus, the present study was aimed to investigate the antinociceptive and cytotoxic activities of methanol and aqueous extracts of *Vanda tessellata* leaf.

## Methods

### Plant material

*Vanda tessellata* leaves were collected from a rustic area (Dholia) of Feni district, Bangladesh and authenticated by the expert of Bangladesh Forest Research Institute, Chittagong, Bangladesh (Accession No. 4306) where a voucher specimen has been deposited for further reference.

### Extract preparation

After washing and cutting the leaves into small parts, the leaves were air-dried in a shade and finally in a mechanical drier (Ecocell, MMM Group, Germany) at 55-60°C. The dried samples were ground to course powder with a mechanical grinder (NOWAKE, Japan). The powder (200 g) soaked in 800 ml methanol for a week at room temperature with occasional shaking and stirring on a shaker machine, and then filtered through a cotton plug followed by whatman filter paper No. 1. The solvent was evaporated under vacuum at room temperature to yield semisolid. To prepare aqueous extract the leaf powder (200 g) was soaked in distilled water (800 ml) overnight at room temperature and the solvent was filtered. The filtrate was distilled and concentrated under reduced pressure. The final yield was 1.6% w/w (VTM) and 1.2% w/w (VTA), respectively. The extracts were preserved in a refrigerator till further use.

### Experimental animals

Swiss albino mice, weighing about 25–30 g, were collected from Jahangir Nagar University, Savar, Bangladesh. The animals were provided with standard laboratory food and distilled water *ad libitum* and maintained at natural day-night cycle having proper ventilation in the room. All the experiments were conducted in an isolated and noiseless condition. The study protocol was approved by the P&D Committee, Department of Pharmacy, International Islamic University Chittagong, Bangladesh (Pharm-P&D-37/07’12). The animals were acclimatized to laboratory condition for 10 days prior to experimentation.

### Drugs and chemicals

Diclofenac Na (MERCK, Mumbai, India), nalbuphine (Square Pharmaceuticals Ltd., Bangladesh), formaldehyde (MERCK, Mumbai, India), and 0.9% NaCl saline solution (Popular Pharmaceuticals Ltd., Bangladesh) were used. All other reagents were of analytical grade.

### Acute oral toxicity test

Acute oral toxicity test was carried out in accordance with the Organization for Economic Cooperation and Development (OECD) guidelines for testing of chemicals, 420 [27]. Swiss albino mice maintained under standard laboratory conditions were used for acute toxicity study. A total of three mice from each group received a single oral dose (5, 50, 300, and 2000 mg/kg bw of the extract. Animals were kept over-night fasting prior to administration. After administration of the extracts (VTA and VTM), food was withheld for further 3 to 4 h. The animals were then individually observed (with special attention during the first 4 h) for possible behavioral changes, allergic reactions (skin rash, itching), eyes and mucous membrane, and mortality for the next 72 h.

### Photochemical screening

For preliminary phytochemical analysis the freshly prepared crude methanol extract of leaves were dissolved in suitable solvent and tested for the presence or absence of phytoconstituents such as reducing sugar, flavonoids, tannins, saponins, phytosterols and alkaloids by using standard phytochemical procedures [28].

### Antinociceptive activity

#### Acetic acid-induced writhing test

For writhing test, 0.6% (v/v) acetic acid solution (10 mL/kg body weight) was injected intraperitoneally (i.p) to each mice of six groups (n = 5) and the number of writhing and stretching were counted over 20 min. Group I served as control received normal saline, Group II received diclofenac sodium (10 mg/kg) as a standard, group III-IV received *V. tessellata* aqueous extract (VTA), and group V-VI received *V. tessellata* methanol extract (VTM) at the dose of 200 and 400 mg/kg bw orally (p.o), respectively 30 min before acetic acid injection [29].

#### Hot plate method

The antinociceptive activity of the extracts was also measured by hot-plate method [30]. Mice were divided into six groups of five animals each. Group I treated as control (saline water 10 mg/kg), group II received standard drug (diclofenac sodium 10 mg/kg i.p), group III-IV received VTA and group V-VI received VTM (200 and 400 mg/kg bw p.o, respectively). The temperature of the hot-plate was maintained at 55 ± 1°C. The mice were placed in a 24 cm diameter glass cylinder on the heated surface and the time between placement and licking of the paws or jumping was recorded as the latency. A cut off time of 20s was followed to avoid any thermal injury to the paws and was defined as complete analgesia. The reaction time were recorded before (0 min) and after 15, 30, 45 and 60 min following administration of test samples or standard drug.

#### Tail immersion method

Animals were screened based on their reaction time at 3–5 sec when subjected to pain stimulus. Mice were divided into six groups of five animals each. Group I represented as control received normal saline and group III-IV received VTA and group V-VI received VTM (200 and 400 mg/kg bw p.o, respectively). Group II received the standard drug nalbuphine (10 mg/kg) subcutaneously (s.c). The animal withdrawing tails from hot water within 5 sec were selected for the study. The lower 3 cm portion of the tail of mice was dipped in a water bath (Le-Chatelier Thermostat, UK) maintaining at temperature of 55 ± 0.5°C. The time in second (s) for tail withdrawal from the water was taken as the reaction time and recoded by a stopwatch at before (0 min) and after 30, 60 and 90 min the administration of test samples. A maximum immersion time of 15 sec was maintained to prevent thermal injury to the animals [31].

#### In vitro cytotoxicity test

General toxicity of the extract was tested by the brine shrimp (*Artemia salina*) lethality assay [32]. Artificial sea water was prepared by dissolving 38 g of NaCl (3.8%) in 1000 ml of distilled water and was filtered off to obtain a clear solution. The dried cysts of the brine shrimps were hatched in artificial seawater with strong aeration for 48 hours. Methanol extract was dissolved in sea water with DMSO (not exceed 0.01%) and transferred to test tubes to obtain concentrations of 12.5, 25, 50, 100, 200 and 400 μg/ml in 5 ml artificial sea water with 20 nauplii in each test tube. Standard drug vincristine sulphate was used as positive control at concentrations of 5, 2.5, 1.25, 0.625, and 0.312 μg/ml. Control test tubes were subjected to DMSO in artificial seawater at the same concentration as in test tubes for test samples. After 24 h incubation at 25-30°C, the number of viable nauplii was counted using a magnifying glass. The percent (%) mortality was calculated using the following formula:


Where, N_t_ = Number of dead nauplii after 24 hrs of incubation, N_0_ = Number of total nauplii transferred (n = 20). The Median lethal concentration (LC_50_) was then determined.

#### Statistical analysis

The data was analyzed by one-way ANOVA followed by *Dunnet’s* test to estimate significant differences between the test and control groups with GraphPad Prism Data Editor for Windows, Version 5.0 (GraphPad software Inc., San Diego, CA). Values were expressed as mean ± Standard error for mean (± SEM). p < 0.05 - 0.01 were considered as statistically significant.

## Results

### Acute oral toxicity test

When a single dose of VTA and VTM was orally administered up to a concentration of 2000 mg/kg bw, no adverse effects and mortality were observed. From the acute oral toxicity test, it can be said that *V. tessellate* has low toxicity profile.

### Phytochemical screening

Phytochemical screening of the leaves extract of *V. tessellata* confirmed the presence of flavonoids, phenolic compounds, tannins and saponins.

### The acetic acid-induced writhing test

The effect of aqueous and methanol extract of *V. tessellata* leaves on acetic acid induced writhing test in mice was dose dependant (Table 1) and significantly (p < 0.05 – P < 0.01) decreased the number of writhing movements induced by the intraperitoneal administration of the acetic acid comparing with positive control. The highest inhibition of pain exhibited by higher dose 400 mg/kg of aqueous and methanol extract was 42.37% and 45.08% respectively while the standard drug, diclofenac Na, was 54.18% (10 mg/kg).

**Table 1:** **Effect of aqueous and methanol extract of** ***V. tessellata*** **leaves in acetic acid-induced writhing test in mice**

Treatment	Dose (mg/kg)	No. of writhing	% of inhibition
Control (Saline water)	10	59.80 ± 1.66	--
Diclofenac Na	10	27.40 ± 2.22**	54.18
VTA	200	41.40 ± 1.63**	30.77
VTA	400	34.46 ± 2.82**	42.37
VTM	200	40.90 ± 0.93*	31.60
VTM	400	32.84 ± 2.23*	45.08

Values are expressed as mean ± SEM; (n = 5). *p < 0.05, **p < 0.01 as compared with control. Data were processed with Dunnet’s test for multiple comparisons, GraphPad Prism for Windows, Version 6.0. VTA = *V. tessellata* aqueous extract, VTM = *V. tessellata* methanol extract.

### Hot plate test

The reaction time of aqueous and methanol extract increased in dose dependant manner to the thermal stimuli. The results are shown in Table 2. The higher dose 400 mg/kg of aqueous and methanol extract was demonstrated the highest nociceptive inhibition of thermal stimulus. The maximum reaction time of aqueous and methanol extracts need for the response against thermal stimuli at higher dose was 13.39 ± 0.64 and 12.61 ± 0.32 seconds at 60 and 45 minutes respectively whereas the diclofenac Na (10 mg/kg) was 16.23 ± 1.66 seconds at 60 minutes (p < 0.01).

**Table 2:** **Effect of aqueous and methanol extract of** ***V. tessellata*** **leaves in hot plate test**

Treatment	Dose (mg/kg)	Reaction time (sec)				
		0 min	15 min	30 min	45 min	60 min
Control (Saline water)	10	3.48 ± 0.58	6.05 ± 0.30	5.43 ± 0.54	5.95 ± 0.87	5.36 ± 0.55
Diclofenac Na	10	4.96 ± 0.34	11.37 ± 1.61*	13.97 ± 0.85**	14.90 ± 1.02*	16.23 ± 1.66**
VTA	200	4.15 ± 0.61	8.66 ± 2.16	9.74 ± 1.41*	11.20 ± 0.96*	11.77 ± 0.81**
VTA	400	3.36 ± 0.88	10.15 ± 1.18*	10.64 ± 2.58	12.73 ± 1.09**	13.39 ± 0.64*
VTM	200	2.91 ± 0.41	9.47 ± 2.00*	9.79 ± 0.89*	10.29 ± 1.52	11.36 ± 0.63*
VTM	400	3.69 ± 0.31	10.68 ± 1.11	11.95 ± 0.85*	12.61 ± 0.32*	12.37 ± 0.51**

Values are expressed as mean ± SEM; (n = 5). *p < 0.05, **p < 0.01 as compared with control. Data were processed with Dunnet’s test for multiple comparisons, GraphPad Prism for Windows, Version 6.0. VTA = *V. tessellata* aqueous extract, VTM = *V. tessellata* methanol extract.

### Tail immersion test

The antinociceptive activity of leaves of *V. tessellata* exhibited in tail immersion test of all two doses significantly increased the latency period of hot water induced thermal stimuli in a dose dependant manner given in Table 3. The highest nociceptive inhibition of thermal stimuli of aqueous and methanol extract at a higher dose 400 mg/kg was 9.91 ± 0.88 and 9.15 ± 1.862 seconds respectively while the inhibition time of nalbuphine was 11.93 ± 1.04 at 10 mg/kg (p < 0.01).

**Table 3:** **Effect of aqueous and methanol extract of** ***V. tessellata*** **leaves in tail immersion test**

Treatment	Dose (mg/kg)	Reaction time (sec)			
		0 min	30 min	60 min	90 min
Control (Saline water)	10	2.21 ± 0.10	2.5 ± 0.18	2.9 ± 0.37	2.39 ± 0.19
Nalbuphine	10	2.27 ± 0.15	6.05 ± 0.49**	9.23 ± 0.40*	11.93 ± 1.04**
VTA	200	2.87 ± 0.40	3.46 ± 0.51*	5.08 ± 0.67	6.38 ± 0.61
VTA	400	2.79 ± 0.34	4.95 ± 0.34	6.38 ± 0.55*	9.91 ± 0.88**
VTM	200	2.16 ± 0.22	2.61 ± 0.10*	5.12 ± 1.66*	4.40 ± 0.53
VTM	400	1.8 ± 0.32	6.2 ± 4.10*	5.43 ± 0.18*	8.15 ± 1.86*

Values are expressed as mean ± SEM; (n = 5). *p < 0.05, **p < 0.01 as compared with control. Data were processed with Dunnet’s test for multiple comparisons, GraphPad Prism for Windows, Version 6.0. VTA = *V. tessellata* aqueous extract, VTM = *V. tessellata* methanol extract.

### The brine shrimp lethality assay

**Figure 1:**
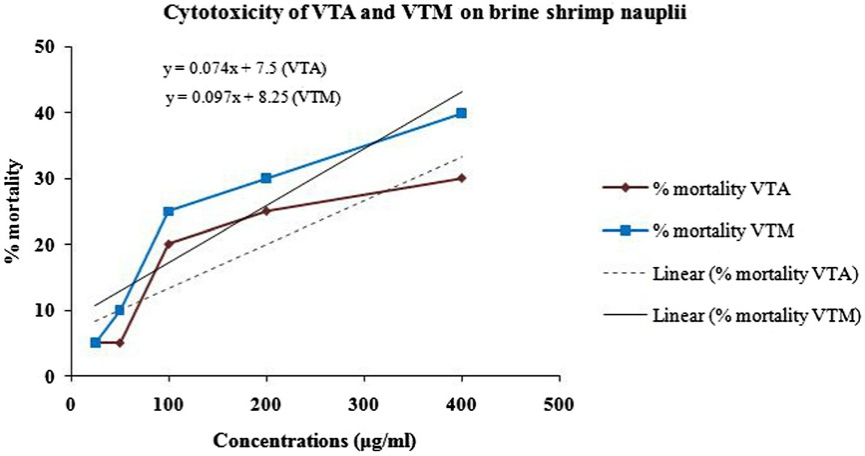
**Effects of various concentrations of** ***V. tessellata*** **leaves on the viability of brine shrimp nauplii after 24 hrs incubation.** VTA = *V. tessellata* aqueous extract, VTM = *V. tessellata* methanol extract.

Cytotoxic effect of the extract is summarized in the Figure 1. The LC_50_ for methanol and aqueous extract of *V. tessellata* leaf were found to be 574.32 and 430.41 μg/mL respectively, and that of vincristine sulphate was 0.74 μg/ml.

## Discussion

The present study evaluated the antinociceptive and cytotoxic effects for the first time of aqueous and methanol leaves extract of *V. tessellata* employing various experimental test models. The study of traditionally used plant species relieving pain should still be seen as a logical research strategy in the search for new analgesic drugs [33, 34].

The intraperitoneal administration of agents provokes a stereotyped behaviour characterised by abdominal contractions and twisting of the dorso-abdominal muscles. This is the advantageous method detecting effects produced by weak analgesics. Writhing test is a non-specific method for evaluation of pain [35]. In this model endogenous mediators such as bradykinin, serotonin and capsaicin generate pain which stimulate peripheral nociceptive neurons by increasing PGE2 and PGF2a [36, 37]. Administration of VTA and VTM exhibited significant (p < 0.05 - p < 0.01) and dose dependent reduction in abdominal contraction compared to control in acetic acid-induced writhing mice. At doses of 200 and 400 mg per kg body weight, the number of writhing, respectively, reduced by 30.77 and 42.37 for VTA, whereas 31.60, and 45.08% for VTM. A standard non-narcotic analgesic drug, diclofenac Na, showed 54.18% inhibition of writhing when administered to experimental animals at doses of 10 mg/kg bw. The extract of *V. tessellata* may act as an inhibitor of prostaglandin synthesis because the nociceptive mechanism of abdominal writhing induced by acetic acid involves the release of arachidonic acid metabolites via cyclooxygenase (COX), and prostaglandin biosynthesis [38]. Furthermore, different flavonoids also act as antinociceptive and anti-inflammatory agents due to their ability to inhibit arachidonic acid metabolism [39–41]. Preliminary phytochemical screening of *V. tessellata* qualitatively identified the presence of flavonoids, phenolic compounds, tannins and saponins. Therefore, flavonoids of the plant might be responsible for antinociceptive activity.

Nociceptive reactions towards thermal stimuli using mice investigate both peripheral and central activity for the detection of opioid analgesic as well as several types of analgesic drug from spinal origin [42–44]. The evaluation of VTA and VTM with the hot plate and tail immersion presented the effect of extract that increased the latency time both doses at 200 and 400 mg/kg after 15 and 30 minutes, respectively. The effect of VTA and VTM at 400 mg/kg doses at 60 and 90 min were significant (p < 0.05) in comparison to control while at dose 200 mg/kg showed fitful significant increase in latency. As the hot plate and tail immersion test is a central antinociceptive test, it proves that extract exerts an antinociceptive effect at least one central mechanisms. Therefore, the findings from chemically and thermally-induced nociceptive processes tested in this study shown by VTA and VTM suggest that the extract contains bioactive compound(s) with central and peripheral antinociceptive actions.

Cytotoxicity assay has been considered as pre-screening assay for antimicrobial, antitumor, antimalarial and insecticidal activities. Therefore it is suggested to be a convenient probe for the pharmacological activities of plant extracts [45, 46]. The brine shrimps lethality assay was used to assess the cytotoxicity of leaves of *V. tessellata*. The LC_50_ value for the crude extracts was found to be very high signifying that the extract is safe at the therapeutic doses.

## Conclusions

Based on obtained results, it can be concluded that *V. tessellata* leaves possess significant and dose-dependent analgesic activity which was validated by various pain models in this study, and has low cytotoxicity. The results substantiate the ethnomedicinal use of *V. tessellata* to palliate pain disorders. The findings of present studies warrant further studies for isolation and identification of the responsible bioactive component(s) and to elucidate the mechanism(s) lying with these effects.

## References

[1] Antman EM, Bennett JS, Daugherty A, Furberg C, Roberts H, Taubert KA (2007). Use of nonsteroidal antiinflammatory drugs: an update for clinicians: a scientific statement from the American heart association. Circulation.

[2] Paulozzi LJ, Ryan GW (2006). Opioid analgesics and rates of fatal drug poisoning in the United States. Am J Prev Med.

[3] Dworkin RH, Backonja M, Rowbotham MC, Allen RR, Argoff CR, Bennett GJ, Bushnell MC, Farrar JT, Galer BS, Haythornthwaite JA, Hewitt DJ, Loeser JD, Max MB, Saltarelli M, Schmader KE, Stein C, Thompson D, Turk DC, Wallace MS, Watkins LR, Weinstein SM (2003). Advances in neuropathic pain: diagnosis, mechanisms, and treatment recommendations. Arch Neurol.

[4] Uprety Y, Asselin H, Boon EK, Yadav S, Shrestha KK (2010). Indigenous use and bio-efficacy of medicinal plants in the Rasuwa District, Central Nepal. J Ethnobiol Ethnomed.

[5] *Medicinal Plants of Bangladesh*. [http://www.mpbd.info/plants/vanda-tessllata.php] (accessed on 14 April, 2014)

[6] Basu K, Das GB, Bhattacharya SK, Lal R, Das PK (1971). Anti-inflammatory principles of *Vanda roxburghii*. Curr Sci.

[7] Ghani A (2003). Medicinal Plants of Bangladesh with Chemical Constituents and Uses.

[8] Kirtikar KR, Basu BD (1975). Indian Medicinal Plants, Plants, Volume 4.

[9] Rahmatullah M, Hasan A, Parvin W, Moniruzzaman M, Khatun A, Khatun Z, Jahan FI, Jahan R (2012). Medicinal plants and formulations used by the Soren clan of the Santal tribe in Rajshahi district, Bangladesh for treatment of various ailments. Afr J Tradit Complement Altern Med.

[10] Hasan M, Akter S, Piya NS, Nath PK, Nova USR, Chowdhury HR, Anjoom NF, Khatun Z, Rahmatullah M (2012). Variations in selection of medicinal plants by tribal healers of the Soren clan of the Santal tribe: a study of the Santals in Rajshahi District, Bangladesh. Am Eurasian J Sustain Agric.

[11] Rahmatullah M, Mollik MAH, Islam MK, Islam MR, Jahan FI, Khatun Z, Seraj S, Chowdhury MH, Islam F, Miajee ZUM, Jahan R (2010). A survey of medicinal and functional food plants used by the folk medicinal practitioners of three villages in Sreepur Upazilla, Magura district, Bangladesh. Am Eurasian J Sustain Agric.

[12] Chawla AS, Sharma AK, Handa SS, Dhar KL (1992). Chemical studies and anti-inflammatory activity of *Vanda roxburghii* roots. Indian J Pharm Sci.

[13] Ahmed F, Sayeed A, Islam A, Salam SA, Sadik G, Sattar MA, Khan GAM (2000). Antimicrobial activity of extracts and a glycoside from *Vanda roxburghii* Br. Pak J Biol Sci.

[14] Rajalakshimi V, Mamola SK, Prapurna S, Rafikhan P, Madhusekhar K (2012). Anti-epileptic activity of *Vanda tessellata* Roxb. on maximal electroshock induced seizure in albino wistar rats. Int J Exp Pharmacol.

[15] Pathan D, Ambavade S (2014). Investigation of anticonvulsant activity of *Vanda roxburghii*. J Pharmacog Phytochem.

[16] Nayak BS, Suresh R, Rao AV, Pillai GK, Davis EM, Ramkissoon V, McRae A (2005). Evaluation of wound healing activity of *Vanda roxburghii* R. Br (Orchidacea): a preclinical study in a rat model. Int J Low Extrem Wounds.

[17] Vijaykumar K (2013). *In vitro* anti-oxidant activity of pet-ether extract of *Vanda tessellata* Roxb. Int Ayur Med J.

[18] Anwar M, Kumar SN, Mahendran B (2013). Hepatoprotective activity of pet-ether extract of *Vanda tessellata* Roxb. Int Ayur Med J.

[19] Teja JN, Pradeep D, Sumanth N, Kumar GV (2012). Anti-diarrhoeal activity of petroleum ether extract of *Vanda tessellata* leaves on castor oil-induced diarrhea in rats. Int J Phytopharm Res.

[20] Sirisha JV, Sailakshmi K, Vijayal K (2013). Human red blood cell (HRBC) membrane stabilizing activity of leaves of pet-ether extract of *Vanda tessellata* Roxb. Int Ayur Med J.

[21] Kumar AS, Gandhimathi R (2012). Protective effect of extract of *Vanda tessellata* against cerebral ischemia in rats. J Pharm Biol.

[22] Kumar PKS, Subramoniam A, Pushpangadan P (2000). Aphrodisiac activity of *Vanda tessellata* (Roxb.) Hook. ex Don extract in male mice. Ind J Pharmacol.

[23] Subramoniam A, Gangaprasad A, Sureshkumar PK, Radhika J, Arun BK (2013). A novel aphrodisiac compound from an orchid that activates nitric oxide synthases. Int J Impot Res.

[24] Rastogi RP, Mehrotra BN (1990). Compendium of Indian Medicinal Plants.

[25] Ahmed F, Sayeed A, Islam A, Salam S, Sadik G, Khan A (2001). Characterization and in vitro antimicrobial activity of 17-β-hydroxy-14,20-epoxy-1-oxo-[22R]-3β-[O-β-d-glucopyranosyl]-5,24-withadienolide from *Vanda roxburghii* Br. J Med Sci.

[26] Uprety Y, Asselin H, Dhakal A, Julien N (2012). Traditional use of medicinal plants in the boreal forest of Canada: review and perspectives. J Ethnobiol Ethnomed.

[27] Organization for Economic Cooperation and Development (OECD) guidelines for testing of chemicals (2001). Acute Oral Toxicity-Fixed Dose Procedure.

[28] Tiwari P, Kumar B, Kaur M, Kaur G, Kaur H (2011). Phytochemical screening and extraction: a review. Int Pharm Sci.

[29] Koster R, Anderson M, Beer DEJ (1959). Acetic acid for analgesic screening. Proc Soc Exp Biol Med.

[30] Eddy NB, Liembach D (1957). Synthetic analgesics II: dithienylbuttenyl and dithiennylbulyl-amines. J Pharmacol Exp Ther.

[31] Singh S, Majumdar DK, Rehan HM (1996). Evaluation of anti-inflammatory potential of fixed oil of *Ocimum sanctum* (Holybasil) and its possible mechanism of action. J Ethnopharmacol.

[32] Mayer BN, Ferrigni NR, Putnam JE, Jacobsen LB, Nichols DE, Mclaughlin JL (1982). Brine shrimp: a convenient bioassay for active plant constituents. Planta Med.

[33] Silva JP, Rodarte RS, Calheiros AS, Souza CZ, Amendoeira FC, Martins MA, Silva PMR, Frutuoso VS, Barreto E (2010). Antinociceptive activity of aqueous extract of *Bowdichia virgilioides* in mice. J Med Food.

[34] de Sá PGS, Nunes XP, de Lima JT, Filho JAS, Fontana AP, de Siqueira J, Quintans-Júnior LJ, Damasceno PKF, Branco CRC, Branco A, da Silva Almeida JRG (2012). Antinociceptive effect of ethanolic extract of *Selaginella convoluta* in mice. BMC Complement Altern Med.

[35] Le Bars D, Gozariu M, Cadden SW (2001). Animal models of nociception. Pharmacol Rev.

[36] Bentley GA, Newton SH, Starr J (1983). Studies on the anti-nociceptive action of agonist drugs and their interaction with opioid mechanisms. Br J Pharmacol.

[37] Hosoi M (1999). Prostaglandin E(2) has antinociceptive effects through EP(1) receptor in the ventromedial hypothalamus in rats. Pain.

[38] Melo MGD, Araújo AAS, Rocha CPL, Almeida EMSA, Siqueira RS, Bonjardim LR, Quintans-Júnior LJ (2008). Purification, physicochemical properties, thermal analysis and antinociceptive effect of atranorin extracted from Cladina kalbii. Biol Pharm Bull.

[39] Middleton E, Kandaswami C, Theoharides TC (2000). The effects of plant flavonoids on mammalian cells: implications for inflammation, heart disease and cancer. Pharmacol Rev.

[40] Havsteen BH (2002). The bioactivity and medical significance of the flavonoids. Pharmacol Ther.

[41] Aquila S, Giner RM, Recio MC, Spegazzini ED, Ríos JL (2009). Anti-inflammatory activity of flavonoids from *Cayaponia tayuya* roots. J Ethnopharmacol.

[42] Vongtau HO, Abbah J, Mosugu O, Chindo BA, Ngazal IE, Salawu AO, Kwanashie HO, Gamaniel KS (2004). Antinociceptive profile of the methanolic extract of *Neorautanenia mitis* root in rats and mice. J Ethnopharmacol.

[43] Headley PM, O’shaughnessy CT (1985). Evidence for opiate and dopamine interaction in striatum. Br J Pharmacol.

[44] Wigdor S, Wilcox GL (1987). Central and systemic morphine-induced antinociception in mice: contribution of descending serotonergic and noradrenergic pathways. J Pharmacol Exp Ther.

[45] Anderson JE, Goetz CM, McLaughlin JL, Suffness M (1991). A blind comparison of simple bench-top bioassays and human tumor cell cytotoxicities as antitumor prescreens. Phytochem Anal.

[46] Rahman MM, Hossain MA, Siddique SA, Biplab KP, Uddin MH (2012). Antihyperglycemic, antioxidant, and cytotoxic activities of *Alocasia macrorrhizos* (L.) rhizome extract. Turk J Biol.

[CR47] The pre-publication history for this paper can be accessed here:http://www.biomedcentral.com/1472-6882/14/464/prepub

